# Training practices in neonatal and paediatric life support: A survey among healthcare professionals working in paediatrics

**DOI:** 10.1016/j.resplu.2020.100063

**Published:** 2021-01-06

**Authors:** Mathijs Binkhorst, Inge M. van der Aar, Marjolein Linders, Arno F.J. van Heijst, Willem P. de Boode, Jos M.T. Draaisma, Marije Hogeveen

**Affiliations:** a Department of Neonatology, Radboud University Medical Center Amalia Children’s Hospital, Nijmegen, The Netherlands; b Department of Paediatrics, Radboud University Medical Center Amalia Children’s Hospital, Nijmegen, The Netherlands

**Keywords:** Survey, Cardiopulmonary resuscitation, Paediatrics, Newborn, Education, Training

## Abstract

**Aim:**

To evaluate neonatal and paediatric life support training practices across Europe.

**Methods:**

We conducted a descriptive study. Paediatric residents, general paediatricians, and subspecialists were surveyed to assess how paediatric basic and advanced life support (PBLS/PALS) and neonatal life support (NLS) are practically arranged and utilised throughout Europe. A mini-Delphi approach was used for survey development. Eligible professionals in general and university hospitals received a web link to the survey.

**Results:**

498 respondents from 16 countries were included. A large majority of responses came from the Netherlands (n = 393) and Belgium (n = 42). Therefore, analysis was based on these responses. PBLS was more frequently offered than PALS and NLS, though not to all professionals caring for children. For PBLS, PALS, and NLS, official recertification varied between 35–75%. Approximately 80–90% had read the latest guidelines, at least partially. Sixty to seventy percent felt capable of instant PALS, 75–90% considered themselves able to perform PBLS and NLS instantly. Not reading the guidelines and less confidence about instant resuscitation seemed to occur more often in the lower and higher age/experience groups compared to the intermediate age/experience groups. A quarter of the respondents <30 years did not feel prepared for instant PALS. General paediatricians appeared to feel most capable of instant resuscitation. General and university hospitals had rather similar training practices and facilities. Manikins were predominantly low-fidelity, especially in general hospitals. Barriers to course participation were high costs, lack of time, the non-compulsory status, remote location, and unavailability of courses.

**Conclusion:**

Although most paediatric professionals receive life support training, guideline reading, recertification, training utilisation, and resuscitation preparedness require improvement. Barriers to course participation should be addressed.

## Introduction

Given the rarity of neonatal and paediatric resuscitations and developments such as restricted duty hours and subspecialisation, many residents and paediatricians are insufficiently exposed to real-life resuscitations to maintain their knowledge and skills regarding basic and advanced life support.^1^, ^2^ To ensure adequate acquisition and retention of resuscitative skills, simulation-based training (SBT) is often employed. It is recommended to participate in such training every 3–6 months.^1^, ^3^, ^4^, ^5^, ^6^ There is evidence that resuscitation performance in the simulation environment correlates with resuscitation competency in the clinical setting, and that life support training improves patient outcomes.^7^, ^8^

The European Resuscitation Council (ERC) issues guidelines for neonatal life support (NLS), paediatric basic life support (PBLS), and paediatric advanced life support (PALS), including recommendations for the education and implementation of these guidelines.^6^, ^9^, ^10^ The information contained in these guidelines is meant to be conveyed to the end users by certified instructors during well-designed resuscitation courses and regular local booster sessions. It is not completely clear to what extent this endeavour is realised in various European countries. Neonatal and paediatric life support training varies considerably among hospitals, institutions, and countries.^2^, ^3^, ^11^, ^12^ We encountered only two large survey studies on cardiopulmonary resuscitation (CPR) training in the literature.^11^, ^12^ One of these pertained to paediatric resuscitation.^12^ Both are not recent and describe the situation from the organisational perspective, detailing about how training is or should be offered. In contrast, we wanted to know how neonatal and paediatric resuscitation training is implemented and utilised in actual practice.

We therefore conducted this survey among paediatric residents, general paediatricians, and paediatric subspecialists (paediatricians with a subspecialty, e.g. paediatric cardiologists, pulmonologists, nephrologists) working in diverse hospitals throughout Europe, to become informed about the reality of life support training from the end users’ point of view. This information may be valuable for resuscitation councils to further optimise resuscitation training for paediatric professionals.

## Methods

In order to describe the methodology of our online survey with sufficient detail, we reported our study according to the recommendations provided in the Checklist for Reporting Results of Internet E-Surveys (CHERRIES) statement.^13^

### Survey development

A multicentre, multidisciplinary, mini-Delphi approach was used to develop our survey. The first draft was created by MB and ML. Items mainly covered the following aspects of neonatal and paediatric life support training: availability, (barriers to) utilisation, compulsory status, design, contents, participants, duration, amount of hands-on practice, equipment and facilities, guideline reading, knowledge assessment, recertification, effects of training on resuscitation preparedness, and ideas about optimal training intervals. The survey consisted of five parts. In the first part, background characteristics were inventoried. The remaining four parts contained items on PBLS, PALS, NLS, and general aspects of training. We used the acronym PALS as an umbrella term to refer to all courses and training modalities in which advanced life support for children is taught. A few items were derived from the aforementioned surveys and from the 2015 ERC guideline.^6^, ^11^, ^12^

The preliminary survey was sent to the mini-Delphi panel, composed of 1 Belgian and 7 Dutch experts in neonatal and paediatric resuscitation (training), and 2 experts in medical education and test development. All were requested to comment on the survey’s structure and contents. Items had to be highlighted in green, orange, or red when they were considered relevant, possibly relevant, or irrelevant, respectively. The sequence and phrasing of the items could be adjusted and additional items could be suggested. MB, IvdA, ML, and MH amended the survey according to the provided feedback. The final survey consisted of 13 pages with a total of 52 items, divided over the subheadings *Background information, PBLS, PALS, NLS,* and *General items*, and was approved by the mini-Delphi panel (Electronic Supplementary material 1). The survey was written in English for all countries. In the introduction section at the beginning of the survey, the investigators were introduced, the study aims were mentioned, statements regarding the (anonymous) handling of the data were made, and an estimate of the time needed to complete the survey (∼10 min.) was given. Items were offered in a fixed order. There were no mandatory items. Some items could be skipped in case they were ‘not applicable’, which could affect the duration and completeness of the survey. Respondents were able to review and change their answers by using the ‘Back’ button. A completeness check was not possible.

Prior to survey dissemination, feasibility testing was performed by MB, ML, and MH. We thereby ensured the usability and technical functionality of our survey.

### Survey distribution and collection

We used SurveyMonkey (©1999–2018, San Mateo, CA 94403, USA) to distribute our survey. Since our survey was not sent to known addressees, but forwarded to as many residents and paediatricians as possible, a web link was created for dissemination. Inasmuch as the questionnaire was only accessible with this link, it was a ‘closed survey’. Considering the confidentiality of certain items, we wanted to guarantee the anonymity of our respondents. In this way, we hoped that respondents felt free to give honest responses. SurveyMonkey removed all identifiable information (including the IP address) from the returned surveys. No personal data were stored. We were only informed about the nationality of our respondents. Although cookies were used, duplicate entries by the same person could theoretically occur, considering the browser-specific nature of the cookies.

### Participant recruitment

We endeavoured to include a broad range of participants and therefore invited general paediatricians, paediatric residents, and subspecialists from both general and university hospitals in countries with different socio-economic circumstances across Europe. We were not able to obtain a suitable database of paediatric professionals. This was mainly due to the confidentiality of personal data held by various councils and organisations, and because the composition of existing databases did not befit our study purposes. Consequently, we constructed our own database. Using phone numbers and email addresses found on the Internet, we contacted the paediatric departments of 162 hospitals in 14 different European countries. For each hospital, we identified a contact person, to whom we could send the web link, with the request to forward it to all general paediatricians, residents, and subspecialists at their own department and in affiliated hospitals (Survey announcement/invitational email in Electronic Supplementary material 2). In addition, we used our contacts (9 paediatricians/neonatologists from 8 different countries) within the Screening to improve Health In Preterm InfantS in Europe (SHIPS) network and (1 Dutch representative of) the Young European Association of Paediatrics (EAP) to forward our survey to as many paediatric colleagues as possible. The study population thus constituted a convenience sample. Participation was voluntary, no incentives were offered to the respondents for completing the survey.

The first email containing the web link was sent in January 2018. A first and second reminder were sent in March and April 2018, respectively. The deadline for response was June 1st, 2018. In the Netherlands, 36 general and 8 university hospitals were addressed; in Belgium 22 and 4, respectively. In the other countries, the ratio of general and university hospitals varied and was mainly dependent on the availability of usable email addresses on the Internet.

### Data analysis

SurveyMonkey collected all responses and generated response percentages and bar charts for all items. For further analysis, we exported all data to Microsoft Excel (version 2007, Microsoft, Redmond, WA, USA). All questionnaires were included in the analysis, irrespective of completeness. Questionnaires were not excluded based on particular timestamps.^13^ Considering the type of data and the inherent occurrence of missing data in such a large-scale survey, we only used descriptive statistics to report our results. In keeping with this, it should be emphasised that statements about how the results of a particular group of respondents related to another are purely meant to highlight some noticeable findings across certain subgroups. These statements solely describe numerical trends and are not based on established statistical differences.

Our anonymous survey was given an exempt determination by the Institutional Review Board of the Radboudumc (file number 2020–7108), since human subjects were not exposed to medical interventions. Respondents automatically consented to participate by returning their completed surveys.

## Results

We included 498 respondents. The response rate could not be determined, since the total number of survey recipients was unknown. The completeness rate of the returned surveys was 79%.^13^ Data were incomplete/missing, because respondents unintentionally skipped items, they rightfully skipped items that were ‘not applicable’ to them, or they did not complete the entire survey. The average time spent on the survey was 7 min.

The number and background characteristics of the respondents from the Netherlands, Belgium, and the other countries are presented in Table 1. In spite of our recruitment efforts, the number of respondents from the other countries was very small. Their responses to the items on PBLS, PALS, and NLS are still presented for completeness (Electronic Supplementary material 3). However, as the results from the other countries probably lacked representativeness, we decided to focus exclusively on the more representative responses from the Netherlands and Belgium in describing our results, also for the general items.

**Table 1:** Background characteristics.

Respondent characteristic	Number (%)		
	Netherlands (n = 393)	Belgium (n = 42)	Other countries (n = 63) a
**Age**			
20−29 years	74 (18.8%)	10 (23.8%)	9 (14.3%)
30−39 years	135 (34.4%)	14 (33.3%)	28 (44.4%)
40−49 years	92 (23.4%)	8 (19.1%)	10 (15.9%)
50–59 years	66 (16.8%)	9 (21.4%)	6 (9.5%)
≥60 years	21 (5.3%)	1 (2.4%)	5 (7.9%)
Missing data	5 (1.3%)	0 (0%)	5 (7.9%)
**Sex**			
Male	101 (25.7%)	9 (21.4%)	19 (30.2%)
Female	287 (73.0%)	33 (78.6%)	40 (63.5%)
Missing data	5 (1.3%)	0 (0%)	4 (6.3%)
**Current function**			
Paediatric resident	149 (37.9%)	14 (33.3%)	27 (42.9%)^b^
General paediatrician	122 (31.0%)	9 (21.4%)	8 (12.7%)
Paediatric subspecialist	120 (30.5%)	19 (45.2%)	23 (36.5%)
Missing data	2 (0.5%)	0 (0%)	5 (7.9%)
**Experience in paediatrics**^c^			
<1 year	26 (6.6%)	2 (4.8%)	2 (3.2%)
1−5 years d	100 (25.4%)	10 (23.8%)	24 (38.1%)^b^
6−10 years	82 (20.8%)	9 (21.4%)	12 (19.0%)
11–20 years	102 (26.0%)	9 (21.4%)	10 (15.9%)
>20 years	82 (20.9%)	11 (26.2%)	11 (17.5%)
Missing data	1 (0.3%)	1 (2.4%)	4 (6.3%)
**Current work place**			
General hospital	209 (53.2%)	15 (35.7%)	15 (23.8%)
University hospital	177 (45.0%)	26 (61.9%)^e^	42 (66.7%)^e^
Other	5 (1.3%) f	1 (2.4%) g	2 (3.2%) h
Missing data	2 (0.5%)	0 (0%)	4 (6.3%)
**Number of beds in institution**			
<500^d^	137 (34.9%)	12 (28.6%)	39 (61.9%)
500−1000	72 (18.3%)	14 (33.3%)	6 (9.5%)
>1000 d	55 (14.0%)	6 (14.3%)	6 (9.5%)
Don’t know	124 (31.5%)	10 (23.8%)	8 (12.7%)
Missing data	5 (1.3%)	0 (0%)	4 (6.3%)
**Life support instructor**			
Certified instructor^i^	55 (14.0%)	7 (16.7%)	14 (22.2%)
Local instructor	47 (12.0%)	8 (19.0%)	8 (12.7%)
No	291 (74.0%)	27 (64.3%)	39 (61.9%)
Missing data	0 (0%)	0 (0%)	2 (3.2%)

a Austria (10), Croatia (3), Denmark (3), Estonia (5), Germany (14), Hungary (1), Ireland (1), Italy (1), Latvia (1), Poland (12), Romania (2), Sweden (1), Switzerland (2), UK (1), Unknown (6).

b Relatively high number of residents – presumably the ones with 1−5 years of experience – in ‘other countries’ probably reflects mediation of Young EAP in participant recruitment.

c Including residency for paediatricians/subspecialists.

d For conciseness, some answer options were grouped together.

e Larger representation of university hospitals in Belgium and ‘other countries’, probably because these hospitals were easier to contact.

f Both general and university hospital (3), general hospital with neonatal intensive care unit (1), research facility (1).

g Both general and university hospital (1).

h Institute (1), ambulatory care facility (1).

i Completed a generic instructor’s course.

Responses to the items on PBLS, PALS, and NLS are shown in Table 2a, Table 2b for the Netherlands and Belgium, respectively. For conciseness, a few straightforward items on verifiable course contents (i.e. more or less factual information about the course programme) were left out from analysis. A minority of the hospitals offered PBLS to all professionals caring for children (including nurses, medical interns, paediatric surgeons, physical therapists, etc.). PBLS was apparently more often offered than PALS and NLS. The recertification rate varied between ∼35−75% for the three types of training. Most respondents (∼80–90%) had read the latest PBLS, PALS, and NLS guidelines, at least partially. Approximately 75–90% considered themselves capable of performing PBLS and NLS instantly; this percentage was ∼60−70% for PALS. Low-fidelity manikins were purportedly more often used than high-fidelity ones.

**Table 2a:** Responses of Dutch participants (n = 393) to items on PBLS, PALS, and NLS.

Item (n)^a^	Responses, n (%)					
**Paediatric basic life support**						
Do you receive	≥1x/year	<1x/year	No/not yet			
PBLS training? (n = 345)	241 (69.9%)	66 (19.1%)	38 (11.0%)			
Does your hospital	Yes	No	Don’t know			
offer PBLS training? (n = 334)	298 (89.2%)	16 (4.8%)	20 (6.0%)			
PBLS training for all professionals	Yes	Most, not all	P, PR and PN	P and PR	Don’t know	Other^b^ 11 (3.4%)
caring for children in your	106 (32.9%)	73 (22.7%)	51 (15.8%)	7 (2.2%)	74 (23.0%)	
hospital? (n = 322)						
Use of high or low-fidelity	High	Low	Both	Neither	Don’t know	
manikins? (n = 321)	33 (10.3%)	151 (47.0%)	100 (31.2%)	5 (1.6%)	32 (10.0%)	
Do you recertify for PBLS as needed? (n = 329)	Yes	No	No guideline^c^	Don’t know		
	223 (67.8%)	55 (16.7%)	7 (2.1%)	44 (13.4%)		
Have you read latest PBLS guideline?^d^ (n = 333)	Completely	Partially	No			
	214 (64.3%)	54 (16.2%)	65 (19.5%)			
Fully capable of performing	Yes	No	Other^e^			
PBLS instantly? (n = 333)	290 (87.1%)	28 (8.4%)	15 (4.5%)			

**Paediatric advanced life support**						
Do you receive	≥1x/year	<1x/year	No/not yet			
PALS training? (n = 332)	182 (54.8%)	103 (31.0%)	47 (14.2%)			
Does your hospital offer PALS training? (n = 310)	Yes	No	Don’t know			
	240 (77.4%)	51 (16.5%)	19 (6.1%)			
Is this PALS training multidisciplinary? (n = 276)	Yes	No	Don’t know			
	183 (66.3%)	68 (24.6%)	25 (9.1%)			
Use of high or low-fidelity manikins? (n = 276)	High	Low	Both	Neither	Don’t know	
	36 (13.0%)	111 (40.2%)	91 (33.0%)	5 (1.8%)	33 (12.0%)	
Duration of latest national PALS course? (n = 276)	1 day	2 days	3 days	4 days	Don’t know	Other^f^
	47 (17.0%)	35 (12.7%)	177 (64.1%)	3 (1.1%)	4 (1.4%)	10 (3.6%)
Hands-on time during latest PALS course? (n = 277)	25%	50%	75%	100%	Don’t know	
	5 (1.8%)	91 (32.9%)	145 (52.3%)	7 (2.5%)	29 (10.5%)	
Do you recertify for PALS as needed? (n = 305)	Yes	No	No guideline^c^	Don’t know		
	224 (73.4%)	47 (15.4%)	9 (3.0%)	25 (8.2%)		
Have you read latest PALS guideline?^d^ (n = 307)	Completely	Partially	No			
	189 (61.6%)	62 (20.2%)	56 (18.2%)			
Fully capable of performing PALS instantly? (n = 309)	Yes	No	Other^g^			
	224 (72.5%)	61 (19.7%)	24 (7.8%)			

**Neonatal life support**^h^						
Do you receive NLS training? (n = 322)	≥1x/year	<1x/year	No/not yet			
	183 (56.8%)	90 (28.0%)	49 (15.2%)			
Does your hospital offer NLS training? (n = 304)	Yes	No	Don’t know			
	245 (80.6%)	36 (11.8%)	23 (7.6%)			
Is this NLS training multidisciplinary? (n = 280)	Yes	No	Don’t know			
	185 (66.1%)	60 (21.4%)	35 (12.5%)			
Use of high or low-fidelity manikins? (n = 276)	High	Low	Both	Neither	Don’t know	
	28 (10.1%)	139 (50.4%)	69 (25.0%)	3 (1.1%)	37 (13.4%)	
Duration of latest national NLS course? (n = 260)	1 day	2 days	3 days	4 days	Don’t know	Other^i^
	219 (84.2%)	23 (8.8%)	0 (0.0%)	0 (0.0%)	6 (2.3%)	12 (4.6%)
Hands-on time during latest NLS course? (n = 263)	25%	50%	75%	100%	Don’t know	
	9 (3.4%)	95 (36.1%)	119 (45.2%)	8 (3.0%)	32 (12.2%)	
Do you recertify for NLS as needed? (n = 306)	Yes	No	No guideline^c^	Don’t know		
	173 (56.5%)	76 (24.8%)	15 (4.9%)	42 (13.7%)		
Have you read latest NLS guideline?^d^ (n = 307)	Completely	Partially	No			
	215 (70.0%)	39 (12.7%)	53 (17.3%)			
Fully capable of performing NLS instantly? (n = 308)	Yes	No	Other^j^			
	267 (86.7%)	28 (9.1%)	13 (4.2%)			

NLS, neonatal life support; P, paediatricians; PALS, paediatric advanced life support; PBLS, paediatric basic life support; PN, paediatric nurses; PR, paediatric residents.

a Number of respondents for each item between parentheses; this number was obtained after subtracting skipped and inapplicable items from 393.

b Emergency department nurses and specialists, paediatric surgeons, anaesthesiologists, residents in anaesthesiology, maternity/obstetric ward nurses, midwifes, gynaecologists, and residents in gynaecology.

c Recertification interval not specified in national guidelines.

d European Resuscitation Council guideline (2015) or national guideline.

e In doubt, ‘on paper’, partly, mostly/probably, insufficient clinical exposure to know, after training, with supervision.

f 1-day refresher course, 2-day refresher course, and several invalid responses.

g In doubt, ‘on paper’, partly, mostly/probably, insufficient clinical exposure to know, after training, with supervision, depending on situation/case, except for cardiac arrhythmia cases.

h Survey stated ‘NLS or NALS’, but Neonatal Advanced Life Support (NALS) courses had just started when this survey was conducted, so responses only pertained to NLS.

i All invalid responses.

j In doubt, ‘on paper’, partly, insufficient clinical exposure to know, after training, with supervision, except neonatal intubation.

**Table 2b:** Responses of Belgian participants (n = 42) to items on PBLS, PALS, and NLS.

Item (n)^a^	Responses, n (%)					
**Paediatric basic life support**						
Do you receive PBLS training? (n = 33)	≥1x/year	<1x/year	No/not yet			
	14 (42.4%)	15 (45.5%)	4 (12.1%)			
Does your hospital offer PBLS training? (n = 31)	Yes	No	Don’t know			
	25 (80.6%)	5 (16.1%)	1 (3.2%)			
PBLS training for all professionals caring for children in your hospital? (n = 29)	Yes	Most, not all	P, PR, and PN	P and PR	Don’t know	Other 0 (0.0%)
	9 (31.0%)	8 (27.6%)	5 (17.2%)	1 (3.4%)	6 (20.7%)	
Use of high or low-fidelity manikins? (n = 29)	High	Low	Both	Neither	Don’t know	
	6 (20.7%)	17 (58.6%)	4 (13.8%)	0 (0.0%)	2 (6.9%)	
Do you recertify for PBLS as needed? (n = 31)	Yes	No	No guideline^b^	Don’t know		
	18 (58.1%)	4 (12.9%)	7 (22.6%)	2 (6.4%)		
Have you read latest PBLS guideline?^c^ (n = 31)	Completely	Partially	No			
	22 (71.0%)	6 (19.4%)	3 (9.7%)			
Fully capable of performing PBLS instantly? (n = 32)	Yes	No	Other			
	29 (90.6%)	3 (9.4%)	0 (0.0%)			

**Paediatric advanced life support**						
Do you receive PALS training? (n = 30)	≥1x/year	<1x/year	No/not yet			
	6 (20.0%)	17 (56.7%)	7 (23.3%)			
Does your hospital offer PALS training? (n = 26)	Yes	No	Don’t know			
	12 (46.2%)	12 (46.2%)	2 (7.7%)			
Is this PALS training multidisciplinary? (n = 19)	Yes	No	Don’t know			
	10 (52.6%)	6 (31.6%)	3 (15.8%)			
Use of high or low-fidelity manikins? (n = 19)	High	Low	Both	Neither	Don’t know	
	2 (10.5%)	11 (57.9%)	3 (15.8%)	0 (0.0%)	3 (15.8%)	
Duration of latest national PALS course? (n = 25)	1 day	2 days	3 days	4 days	Don’t know	Other
	2 (8.0%)	18 (72.0%)	5 (20.0%)	0 (0.0%)	0 (0.0%)	0 (0.0%)
Hands-on time during latest PALS course? (n = 23)	25%	50%	75%	100%	Don’t know	
	1 (4.3%)	5 (21.7%)	16 (69.6%)	1 (4.3%)	0 (0.0%)	
Do you recertify for PALS as needed? (n = 25)	Yes	No	No guideline^b^	Don’t know		
	16 (64.0%)	3 (12.0%)	4 (16.0%)	2 (8.0%)		
Have you read latest PALS guideline?^c^ (n = 25)	Completely	Partially	No			
	19 (76.0%)	4 (16.0%)	2 (8.0%)			
Fully capable of performing PALS instantly? (n = 25)	Yes	No	Other^d^			
	15 (60.0%)	7 (28.0%)	3 (12.0%)			

**Neonatal life support**^e^						
Do you receive NLS training? (n = 27)	≥1x/year	<1x/year	No/not yet			
	8 (29.6%)	10 (37.0%)	9 (33.3%)			
Does your hospital offer NLS training? (n = 26)	Yes	No	Don’t know			
	20 (76.9%)	3 (11.5%)	3 (11.5%)			
Is this NLS training multidisciplinary? (n = 22)	Yes	No	Don’t know			
	15 (68.2%)	4 (18.2%)	3 (13.6%)			
Use of high or low-fidelity manikins? (n = 22)	High	Low	Both	Neither	Don’t know	
	1 (4.5%)	13 (59.1%)	3 (13.6%)	0 (0.0%)	5 (22.7%)	
Duration of latest national NLS course? (n = 19)	1 day	2 days	3 days	4 days	Don’t know	Other^f^
	16 (84.2%)	1 (5.3%)	0 (0.0%)	0 (0.0%)	0 (0.0%)	2 (10.5%)
Hands-on time during latest NLS course? (n = 18)	25%	50%	75%	100%	Don’t know	
	2 (11.1%)	4 (22.2%)	12 (66.7%)	0 (0.0%)	0 (0.0%)	
Do you recertify for NLS as needed? (n = 25)	Yes	No	No guideline^b^	Don’t know		
	9 (36.0%)	4 (16.0%)	7 (28.0%)	5 (20.0%)		
Have you read latest NLS guideline?^c^ (n = 25)	Completely	Partially	No			
	14 (56.0%)	7 (28.0%)	4 (16.0%)			
Fully capable of performing NLS instantly? (n = 25)	Yes	No	Other			
	19 (76.0%)	6 (24.0%)	0 (0.0%)			

NLS, neonatal life support; P, paediatricians; PALS, paediatric advanced life support; PBLS, paediatric basic life support; PN, paediatric nurses; PR, paediatric residents.

a Number of respondents for each item between parentheses; this number was obtained after subtracting skipped and inapplicable items from 42.

b Recertification interval not specified in national guidelines.

c European Resuscitation Council guideline (2015) or national guideline.

d In doubt, probably, with supervision.

e Survey stated ‘NLS or NALS’, but Neonatal Advanced Life Support (NALS) courses had just started when this survey was conducted, so responses only pertained to NLS.

f Two invalid responses.

Of the certified and local instructors, 10% and 20%, 10% and 24%, and 13% and 13% indicated that they did not completely read the latest PBLS, PALS, and NLS guidelines, respectively. Eight and sixteen percent of the certified and local instructors did not feel fully capable of instant PALS, respectively. Most responses of participants employed at general hospitals appeared to be similar to the responses of participants working in university hospitals, with two exceptions. First, the sense of preparedness for PALS: 74% and 56% of the respondents from general and university hospitals felt prepared for PALS, respectively. Second, the type of manikin used: general hospitals inclined towards the use of low-fidelity manikins (43% basic, 7% advanced, 17% both, 33% neither/‘don’t know’), while university hospitals inclined towards the use of both low-fidelity and high-fidelity manikins (24% basic, 9% advanced, 24% both, 43% neither/‘don’t know’). General paediatricians apparently felt more capable of performing instant PBLS, PALS, and NLS than residents and subspecialists.

In the lower (<30 years/<1–2 years of experience) and higher (≥50 years/>20 years of experience) age/experience groups, less respondents seemed to have read the latest PBLS and PALS guidelines compared to the intermediate age/experience groups. Guideline reading was especially poor in the highest age group: ∼30% did not read the PBLS, PALS, and/or NLS guidelines. The highest percentage of professionals not feeling fully confident about instant PBLS and PALS was reported among respondents <30 years of age with <1-2 years of experience; about a quarter of them did not feel prepared for instant PALS. In all age/experience groups, less than 14% of the respondents indicated to feel unprepared for NLS. Training in non-technical skills occurred, on average, more often with increasing age. Male and female respondents reported that they felt able to perform instant PBLS, PALS, and NLS in 76% and 72%, 59% and 54%, and 73% and 63%, respectively.

Approximately two-thirds of the Dutch and half of the Belgian respondents answered that they have a simulation facility available (Table 3). General and university hospitals were seemingly comparable regarding the availability of a simulation facility. The majority of the respondents indicated that they debrief after (simulated) resuscitations. Video-supported debriefing and e-learnings were apparently not widely implemented. The main barriers for partaking in national resuscitation courses seemed to be high costs, lack of time/other clinical priorities, the non-compulsory status, remote location, and unavailability of courses. Regular participation in local resuscitation training was frequently mentioned as a reason for not participating in national/certified courses. Most respondents considered it necessary to train resuscitation skills at least every year. According to several respondents, the most desirable training schedule would consist of a national course every 2–5 years, interspersed with local booster training with an interval ranging from weekly to every 6 months. Others mentioned that training should ideally be tailored to the specific needs of individuals, depending on their professional role, responsibilities, and clinical exposure.

**Table 3:** Responses (n = 498) to general items.

Item	Responses, n (%)		
	Netherlands (n = 393)	Belgium (n = 42)	Other countries (n = 63)^a^
**Simulation facility available?**			
Yes	258 (65.6%)	20 (47.6%)	29 (46.0%)
No	43 (11.0%)	3 (7.1%)	10 (15.9%)
Don’t know	17 (4.3%)	2 (4.8%)	3 (4.8%)
Missing data	75 (19.1%)	17 (40.5%)	21 (33.3%)
**Video recordings used for feedback?**			
Yes	79 (20.1%)	3 (7.1%)	15 (23.8%)
No	198 (50.4%)	17 (40.5%)	27 (42.9%)
Don’t know	43 (10.9%)	6 (14.3%)	1 (1.6%)
Missing data	73 (18.6%)	16 (38.1%)	20 (31.7%)
**Formal debriefing after…..?**			
Simulated resuscitations	11 (2.8%)	1 (2.4%)	9 (14.3%)
Real-life resuscitations	38 (9.7%)	8 (19.0%)	3 (4.8%)
Both	261 (66.4%)	12 (28.6%)	24 (38.1%)
Neither	8 (2.0%)	5 (11.9%)	7 (11.1%)
Missing data	75 (19.1%)	16 (38.1%)	20 (31.7%)
**Training in non-technical skills?**^b^			
Communication skills	221	15	24
Teamwork	190	12	25
Leadership	154	13	17
Situational awareness	154	11	18
CRM	145	5	12
Other^c^	13	1	4
**Certified e-learnings available?**			
Yes	114 (29.0%)	10 (23.8%)	7 (11.1%)
No	24 (6.1%)	4 (9.5%)	12 (19.1%)
Don’t know	181 (46.1%)	12 (28.6%)	24 (38.1%)
Missing data	74 (18.8%)	16 (38.1%)	20 (31.7%)
**Barriers to course participation?**^b^			
Too busy	153	16	29
Course too expensive	104	14	24
Other priorities	101	11	8
Course not compulsory	49	11	10
Insufficient courses	43	8	15
Course site too far away	56	0	1
Quality course materials too low	22	2	8
Insufficient instructors	13	5	7
Inadequate instructors	7	0	1
Other^d^	23	0	0
No barriers	66	2	5
**Optimal interval for retraining?**			
Every 3 months	72 (18.3%)	3 (7.1%)	7 (11.1%)
Every 6 months	85 (21.6%)	4 (9.5%)	15 (23.8%)
Every year	96 (24.4%)	10 (23.8%)	15 (23.8%)
Every 2 years	39 (9.9%)	9 (21.5%)	6 (9.6%)
Other^e^	25 (6.4%)	0 (0.0%)	0 (0.0%)
Missing data	76 (19.4%)	16 (38.1%)	20 (31.7%)

CRM, crew resource management.

a Austria (10), Croatia (3), Denmark (3), Estonia (5), Germany (14), Hungary (1), Ireland (1), Italy (1), Latvia (1), Poland (12), Romania (2), Sweden (1), Switzerland (2), UK (1), Unknown (6).

b Multiple responses per participant possible; total number of responses not known and percentages not computable.

c No training in non-technical skills (13), invalid response (4), don’t know (1).

d Regular participation in local/in-hospital/on site resuscitation training (9), course duration (4), fear for the final assessment (1), the fact that it is not offered to all staff members (1), logistic difficulties (2); six ‘other’ responses were invalid.

e PALS every 3 years, NLS every year (1), regular on site training (2), interval depends on profession, responsibilities, and clinical exposure (7), a national/certified course every 2–5 years, complemented with local training weekly to every 6 months (15).

## Discussion

This survey provides an overview of neonatal and paediatric resuscitation training practices. Although we endeavoured to describe these practices for various European countries, the small number of respondents from all countries except the Netherlands and Belgium precluded the intended, wide-ranging overview. We therefore focused on the reasonably representative results obtained from the Dutch and Belgian respondents. Most paediatric professionals employed in general and university hospitals regularly attend PBLS, PALS, and NLS training. Both hospital types have simulation facilities, in which low-fidelity manikins are probably used most frequently. It appears that PBLS is offered the most, although usually not to all professionals caring for children (e.g. paediatric surgeons, interns). We think it is prudent to ensure that all professionals, who care for children on a regular basis, are trained in PBLS. A considerable number of paediatric professionals does not officially recertify for PBLS, PALS, and NLS, for which several, mainly extrinsic factors were identified. They are apparently less inclined to attend national/certified courses, when they already engage regularly in local training sessions. Various paediatric professionals, especially the most junior and senior ones, do not (completely) read the guidelines and are not fully confident about performing PBLS, PALS, and NLS *ad hoc*. General paediatricians apparently feel most capable of performing all three types of life support. This ostensibly higher resuscitation readiness in general paediatricians and mid-age professionals may, of course, not only reflect their level of training; it may also be indicative of an increased exposure to resuscitations during their daily work.

Comparing our results to previous studies was difficult, since most related articles were published several years ago.^2^, ^3^, ^11^, ^12^ In the meantime, insights, guidelines, and training practices have changed based on emerging evidence. Also, whereas the aforementioned surveys on CPR training reported information based on the responses of experts, trainers, and organisations,^11^, ^12^ we focused on the practical realisation of resuscitation training as experienced by participants. Furthermore, the results of the most relevant survey were mainly obtained on a different continent, where guidelines, socio-economic circumstances, and logistic challenges are importantly different compared to Europe.^12^ Nonetheless, according to this latter survey, CPR training was offered to paediatric residents in only 26.6% of all countries. This seemed to contrast our findings, since 76%, 67%, and 69% of the residents in our survey received PBLS, PALS, and NLS training, respectively. The other large survey, evaluating (adult) CPR training in Europe, showed that physicians received BLS and/or ALS training in 76% of the surveyed hospitals.^11^ Our respondents indicated that 88%, 78%, and 80% of the hospitals offered PBLS, PALS, and NLS, respectively. In the survey by Garcia-Barbero et al.,^11^ hours spent on theoretical tuition and practical training were comparable, whereas we found that the majority of time during PALS and NLS training was devoted to practical, hands-on training.

Several respondents declared themselves not completely capable of performing neonatal and/or paediatric life support *ad hoc*. Our results seemed to indicate that this sense of unpreparedness was most often experienced by the youngest and most senior professionals. It seems likely that this resuscitation unpreparedness results from a lack of training and clinical exposure, being most pronounced at both ends of these professionals’ career. Especially noteworthy was the finding that a quarter of the youngest respondents did not consider themselves fully capable of PALS. In a study by Gemke et al., residents reported that they experienced more self-confidence and less stress when resuscitating newborns compared to older children.^3^ Our results corroborated this notion, since our participants in general, and the residents in particular, thought that they were more competent in NLS (and PBLS) than in PALS. This is probably due to more exposure to NLS and the more homogenous character of neonatal resuscitation.^3^ Furthermore, females seemed to feel a little less at ease when instantly summoned to a resuscitation scene compared to males (although the significance of this and other differences could not be demonstrated due to the descriptive design of our study). On the one hand, our data may suggest that some subgroups should be (re)trained with priority. On the other hand, since we previously demonstrated a large discrepancy between self-assessed competence in PBLS (51%) and actual performance on an unannounced simulated PBLS examination (21% pass rate),^1^ one may also speculate that males, general paediatricians, and professionals in the intermediate age/experience groups are more inclined to overestimate their capabilities.

In recent years, advanced technological and educational possibilities have become available for resuscitation training, such as high-fidelity manikins, e-learnings, and video debriefing. The ERC considers low-fidelity manikins appropriate for all levels of training.^6^ This survey revealed that these manikins are widely used throughout Europe. A meta-analysis on this topic only showed a small benefit of high-fidelity manikins compared to low-fidelity manikins in improving skill performance at the end of a resuscitation course.^14^ However, there was no benefit for knowledge at course conclusion, skill performance between course conclusion and follow-up at one year, and skill performance one year later. Although high-fidelity manikins are popular with learners, they are more expensive and may even conduce to over-confidence.^15^ Other studies supported these findings and concluded that retention of skills does not improve more when using high-fidelity manikins.^16^, ^17^ Video debriefing and e-learnings are not commonly used in resuscitation training according to our respondents; perhaps they will gain popularity when evidence is accumulated in support of them.^18^, ^19^, ^20^

Although most paediatric professionals adequately recertified for PBLS, PALS, and NLS, some barriers to participation in national/certified courses appeared to exist. These were mainly extrinsic (logistic, organisational, financial) in nature; intrinsic factors (poor quality of course instructors, contents, and materials) seemed to be less of an issue. In previous studies, insufficient instructors and teaching materials, costs, organisational deficiencies, and the non-compulsory status of resuscitation courses were identified as barriers to course participation.^2^, ^12^ Participation in local resuscitation training is often stated as a reason for not attending national/certified (refresher) courses. In-hospital resuscitation training is regarded as a reasonable alternative to keep one’s skills up-to-par. This practice is especially defensible in case of the in-house availability of proper facilities and certified instructors. Decentralisation of resuscitation training may even be formalised in the future by starting official outreach simulation programmes.^21^ Outreach simulation has potential benefits, such as training with the usual multidisciplinary team in a familiar environment, which may prevent difficulties with contextual adaptation, and the possibility of providing feedback on work flow and system errors.^6^, ^22^ Nevertheless, resuscitation councils should endeavour to eradicate the abovementioned barriers to facilitate paediatric professionals to acquire and maintain their official provider status. Successful completion of relevant resuscitation courses may be declared compulsory for the periodic re-registration of paediatricians. In the Netherlands, PBLS, PALS, and NLS are already compulsory components of the residency programme. Reducing the costs of course participation can be achieved by exploitation of peer teaching for nurses and residents, increasing efficiency, saving on the course venue, reducing the duration of instructor-led training by using blended learning approaches, and relying on the demonstrated effectiveness of self-directed learning.^2^, ^6^, ^12^, ^23^ The availability of courses as well as the number and remoteness of course sites may be reconsidered. Also, recertification requirements may be adjusted to the individual needs of professionals, based on their clinical role and exposure. In the end, an optimal combination of high-frequency, low dose in-situ training and low-frequency, high-dose certified courses should be pursued.^6^

### Strengths and limitations

We included a fairly large number of respondents, with a seemingly adequate representation of all intended subgroups. Information was obtained from professionals employed in general and university hospitals. Our respondents are the participants of neonatal and paediatric resuscitation courses. Therefore, their feedback, views, and experiences are really valuable for evaluating how life support training is realised in actual practice. Further strengths were the diligent development of the survey and the anonymous acquisition of the data, which hopefully led to honest instead of socially desirable responses.

Although inevitable in such a large-scale survey, we had a fair amount of missing data. Also, the descriptive nature of our study precluded significance testing for potential differences. We nevertheless think that our results provide valuable insights. Since we were only informed about the nationality of our respondents, it was theoretically possible that various respondents from one country all worked in the same hospital. Also, duplicate entries from the same individual could not be completely ruled out, but were deemed unlikely. Due to the voluntary nature of this survey, a selection bias (volunteer effect) could have occurred, for respondents interested in resuscitation training may have been more inclined to participate. This may have caused an overestimation of some of our results. Finally, the poor representation of the other European countries prevented us from obtaining a more comprehensive impression of training practices in Europe.

## Conclusions

Most paediatric professionals in the Netherlands and Belgium receive regular resuscitation training, mainly PBLS. Guideline reading and resuscitation preparedness appear to be suboptimal, especially among the youngest and most senior professionals. PALS training for residents probably needs to be optimised. Advanced technologies (high-fidelity manikins, video-based debriefing, e-learnings) are not yet widely applied in paediatric resuscitation training. Retraining and recertification should be done at set intervals, although it is open to debate whether this can be individualised and decentralised. Barriers to course participation should be removed to facilitate training for all paediatricians and residents. Future studies, ideally performed by an international collaboration, are needed to collect more robust data on the training practices of paediatric professionals in Europe and other parts of the world.

## Conflicts of interest

M. Hogeveen and J. Draaisma are instructor and course director of the Newborn (Advanced) Life Support and European Paediatric Advanced Life Support courses of the Dutch Foundation for the Emergency Medical Care of Children, respectively. The other authors have no ethical or financial conflicts of interest.

## Funding source

This study was carried out without funding.

## Ethics and patient consent

Our anonymous survey was given an exempt determination by the Institutional Review Board of the Radboudumc, since human subjects were not exposed to medical interventions. Respondents automatically consented to participate by returning their completed surveys.

## Data sharing statement

Data collected in this study, but not presented in this manuscript, can be obtained from the authors on request.

## Author contributions

All authors made significant contributions to the conceptualisation and design of the study as well as the acquisition, analysis, and interpretation of the data. MB, IvdA, and ML were responsible for drafting the initial manuscript. AvH, WdB, JD, and MH critically revised the manuscript for important intellectual content. All authors approved the final version of the manuscript as submitted.
