# Supplementary material for: Training practices in neonatal and paediatric life support: A survey among healthcare professionals working in paediatrics
Source: Resusc Plus. 2021 Jan 6;5:100063. doi: 10.1016/j.resplu.2020.100063 (PMC8244515; doi:10.1016/j.resplu.2020.100063)
Supplement: Supplementary file 1 [file mmc1.pdf]

European survey

Dear colleague,

This large European survey is part of a Dutch research project titled *Paediatric and neonatal life support: teaching, training, and testing.* The survey is designed with the help of various experts in the fields of paediatric and neonatal life support and medical education. It is meant to be completed by **paediatricians (with or without subspecialty) and residents in paediatrics** . Our goal is to gain insight in the implementation and application of paediatric and neonatal resuscitation guidelines and to evaluate how teaching, training, and testing of paediatric and neonatal life support actually take place in your hospital and country.

We kindly ask you to complete our survey, which will take approximately **10 minutes**. Please report us honestly how things are arranged in daily practice, not how they should be in the ideal situation. All respondents and hospitals will remain **anonymous**. Data will only be reported per country.

We truly appreciate your valuable contribution to our research!

On behalf of all co-workers,

Mathijs Binkhorst, MD, PhD student
Paediatrician-neonatologist
Radboud University Medical Centre
Amalia Children's Hospital
Nijmegen, The Netherlands

Background information

Please provide us some information about your background by answering the questions below.

1. Age

- 20-29 years
- 30-39 years
- 40-49 years
- 50-59 years
- ≥ 60 years

2. Sex

- Male
- Female

3. Current function

- Paediatric resident
- General paediatrician
- Paediatric subspecialist

4. Experience in paediatrics (including residency)

- < 1 year
- 1-2 years
- 3-5 years
- 6-10 years
- 11-20 years
- > 20 years

5. I currently work in a .....

- University hospital
- General hospital
- Other (please specify)

6. Number of beds in your hospital/institution

- < 100
- 100-500
- 500-1000
- 1000-1500
- > 1500
- Don't know

7. Country

- Belgium
- Denmark
- Estonia
- France
- Germany
- Greece
- Italy
- Luxembourg
- The Netherlands
- Poland
- Romania
- Sweden
- Switzerland
- United Kingdom
- Other (please specify)

8. Are you a paediatric and/or neonatal life support instructor?

- Yes, I completed a generic instructor's course
- Yes, I am a local instructor, but not officially certified
- No

Paediatric Basic Life Support (PBLS)

Paediatric Basic Life Support (PBLS) is defined as airway, breathing, and circulation support to restore or maintain vital functions in a child, without the use of medical equipment other than a protective device and the use of an automated external defibrillator.

Note: **course** means an official/national/certified resuscitation course. **Training** also refers to other sorts of resuscitation education, such as hospital-based simulation training, in-situ team training, bedside booster sessions, and other examples of local initiatives to ensure acquisition and retention of knowledge and/or skills regarding paediatric resuscitation.

9. Do you receive training in Paediatric Basic Life Support (PBLS)?

- ○ Yes, at least once a year
- ○ Yes, less than once a year
- ○ No / Not yet (skip the remaining questions on PBLS and move on to the next page on PALS)

10. Does your hospital/department offer PBLS training?

- ○ Yes
- ○ No (skip questions 11 and 12)
- ○ Don't know (skip questions 11 and 12)

For the next question
Examples of healthcare providers that come into contact with children are: paediatric surgeons, paediatric radiologists, paediatric physical therapists, paediatric nurses, and medical interns.

11. Is PBLS training offered to all healthcare providers that come into contact with children in your hospital?

- ○ Yes, all healthcare providers that come into contact with children are trained in PBLS
- ○ Most, but not all healthcare providers that come into contact with children are trained in PBLS
- ○ No, PBLS training is only provided to paediatricians, paediatric residents, and paediatric nurses
- ○ Other (please specify)
- ○ No, PBLS training is only provided to paediatricians and paediatric residents
- ○ Don't know

For the next question
Basic manikins are non-recording, low-fidelity manikins. Examiners have to rely on visual assessment to evaluate compressions and ventilations.
Advanced manikins are recording, high-fidelity manikins that provide feedback on compressions and ventilations.

12. Are basic or advanced manikins used for PBLS training in your hospital?

- ◯ Basic manikins
- ◯ Advanced manikins
- ◯ Both basic and advanced manikins
- ◯ Neither basic nor advanced manikins
- ◯ Don't know

13. Did you learn to use an Automated External Defibrillator (AED) during PBLS training?

- ◯ Yes
- ◯ No
- ◯ Don't know

14. Do you recertify for PBLS according to national guidelines (i.e. every so many years)?

- ◯ Yes
- ◯ No
- ◯ There are no national guidelines for PBLS recertification
- ◯ Don't know

15. Is PBLS part of the official/national Paediatric Advanced Life Support (PALS) course?

- ◯ Yes
- ◯ No
- ◯ Don't know

16. Is a standardized theoretical PBLS exam (knowledge test) available in your country?

- ◯ Yes
- ◯ No
- ◯ Don't know

17. Have you read the latest (2015) European Resuscitation Council (ERC) or national guideline on PBLS?

- ◯ Yes
- ◯ Partially
- ◯ No

18. Do you consider yourself fully capable of performing PBLS instantly?

- ◯ Yes
- ◯ No
- ◯ Other (please specify)

Paediatric Advanced Life Support (PALS)

First note: **course** means an official/national/certified resuscitation course. **Training** also refers to other sorts of resuscitation education, such as hospital-based simulation training, practicing ABCDE-scenarios, in-situ team training, bedside booster sessions, and other examples of local initiatives to ensure acquisition and retention of knowledge and/or skills regarding paediatric resuscitation.

Second note: for Dutch participants, this section refers to Advanced Paediatric Life Support (APLS).

19. Do you receive training in Paediatric Advanced Life Support (PALS)?

- ◯ Yes, at least once a year
- ◯ Yes, less than once a year
- ◯ No / Not yet (skip the remaining questions on PALS and move on to the next page on NLS/NALS)

20. Does your hospital/department offer PALS training?

- ◯ Yes
- ◯ No (skip questions 21, 22, and 23)
- ◯ Don't know (skip questions 21, 22, and 23)

For the next question
Multidisciplinary means, for example, paediatricians, nurses, anesthesiologists, and/or emergency care physicians training together.

21. Is PALS training multidisciplinary in your hospital?

- ◯ Yes
- ◯ No
- ◯ Don't know

For the next question
Basic manikins are non-recording, low-fidelity manikins. Examiners have to rely on visual assessment to evaluate compressions and ventilations.
Advanced manikins are recording, high-fidelity manikins that provide feedback on compressions and ventilations.

22. Are basic or advanced manikins used for PALS training in your hospital?

- ◯ Basic manikins
- ◯ Advanced manikins
- ◯ Both basic and advanced manikins
- ◯ Neither basic nor advanced manikins
- ◯ Don't know

23. Do paediatric nurses, who work at your department, receive periodic compulsory PALS training?

- Yes
- No
- Don't know

24. Did you learn to use a defibrillator during PALS training?

- Yes
- No
- Don't know

25. Did you learn to intubate a child during PALS training?

- Yes
- No
- Don't know

26. Did you learn to place an intraosseous access during PALS training?

- Yes
- No
- Don't know

27. What was the duration of your latest official/national PALS course?

- 1 day
- 2 days
- 3 days
- 4 days
- Don't know
- Not applicable
- Other (please specify)

28. During your latest PALS course, what was the approximate percentage of time devoted to hands-on practice?

- 25%
- 50%
- 75%
- 100%
- Don't know
- Not applicable

29. Do you recertify for PALS according to national guidelines (i.e. every so many years)?

- ○ Yes
- ○ No
- ○ There are no national guidelines on PALS recertification
- ○ Don't know

30. Is a standardized theoretical PALS exam (knowledge test) available in your country?
Such a knowledge test may be used during the official/national PALS course.

- ○ Yes
- ○ No
- ○ Don't know

31. Have you read the latest (2015) European Resuscitation Council (ERC) or national guideline on PALS?

- ○ Yes
- ○ Partially
- ○ No

32. Do you consider yourself fully capable of performing PALS instantly?

- ○ Yes
- ○ No
- ○ Other (please specify)

[ ]

Neonatal (advanced) life support (NLS/NALS)

First note: **course** means an official/national/certified resuscitation course. **Training** also refers to other sorts of resuscitation education, such as hospital-based simulation training, in-situ team training, bedside booster sessions, and other examples of local initiatives to ensure acquisition and retention of knowledge and/or skills regarding paediatric resuscitation.

Second note: in the questions below, **NLS may also be read as NALS**.

33. Do you receive training in neonatal life support (NLS)?

- ◯ Yes, at least once a year
- ◯ Yes, less than once a year
- ◯ No / Not yet (skip the remaining questions on NLS and move on to the next page with General questions)

34. Does your hospital/department offer NLS training?

- ◯ Yes
- ◯ No (skip questions 35, 36, and 37)
- ◯ Don't know (skip questions 35, 36, and 37)

For the next question
Multidisciplinary means, for example, neonatologists, nurses, obstetricians, and/or anesthesiologists training together.

35. Is NLS training multidisciplinary in your hospital?

- ◯ Yes
- ◯ No
- ◯ Don't know

For the next question
Basic manikins are non-recording, low-fidelity manikins. Examiners have to rely on visual assessment to evaluate compressions and ventilations.
Advanced manikins are recording, high-fidelity manikins that provide feedback on compressions and ventilations.

36. Are basic or advanced manikins used for NLS training in your hospital?

- ◯ Basic manikins
- ◯ Advanced manikins
- ◯ Both basic and advanced manikins
- ◯ Neither basic nor advanced manikins
- ◯ Don't know

37. Do nurses, who work on the neonatal (sub)unit of your department, receive periodic compulsory NLS training?

- Yes
- No
- Don't know

38. Did you learn to intubate a newborn during NLS training?

- Yes
- No
- Don't know

39. Did you learn to insert an umbilical venous catheter during NLS training?

- Yes
- No
- Don't know

40. What was the duration of your latest official/national NLS course?

- 1 day
- 2 days
- 3 days
- Other (please specify)
- 4 days
- Don't know
- Not applicable

41. During your latest NLS course, what was the approximate percentage of time devoted to hands-on practice?

- 25%
- 50%
- 75%
- 100%
- Don't know
- Not applicable

42. Do you recertify for NLS according to national guidelines (i.e. every so many years)?

- Yes
- No
- There are no national guidelines for NLS recertification
- Don't know

43. Is a standardized theoretical NLS exam (knowledge test) available in your country?
Such a knowledge test may be used during the official/national NLS course.

- Yes
- No
- Don't know

44. Have you read the latest (2015) European Resuscitaton Council (ERC) or national guideline on NLS?

- Yes
- Partially
- No

45. Do you consider yourself fully capable of performing NLS instantly?

- Yes
- No
- Other (please specify)

General questions

Note: course means an official/national/certified resuscitation course. Training also refers to other sorts of resuscitation education, such as hospital-based simulation training, in-situ team training, bedside booster sessions, and other examples of local initiatives to ensure acquisition and retention of knowledge and/or skills regarding paediatric resuscitation.

46. Is a simulation facility available for resuscitation training in your hospital?

- ◯ Yes
- ◯ No
- ◯ Don't know

47. Is video feedback used for the examination of paediatric and/or neonatal life support in your hospital?

- ◯ Yes
- ◯ No
- ◯ Don't know

48. Do you formally debrief with your team members after......?

- ◯ Simulated resuscitations
- ◯ Real-life resuscitations
- ◯ Both
- ◯ Neither

49. Do you receive training in the following non-technical resuscitation skills? More than one answer possible.

- ☐ Communication skills
- ☐ Situational awareness
- ☐ Leadership
- ☐ Crisis resource management (CRM)
- ☐ Teamwork
- ☐ Other (please specify)

[ ]

50. Are certified e-learnings available in your country to study paediatric and/or neonatal life support?

- ◯ Yes
- ◯ No
- ◯ Don't know

51. In your opinion, what are the main obstacles to improve the attendance at and quality of paediatric and neonatal resuscitation courses? More than one answer possible.

- ☐ Paediatricians/residents are often too busy with other tasks
- ☐ Paediatricians/residents give priority to other tasks
- ☐ The course site is too far away
- ☐ The courses are too expensive
- ☐ The courses are not compulsory for paediatricians/residents
- ☐ The quality of the training materials (e.g. manikins, equipment, course manual, presentations) is too low
- ☐ The course instructors are not fully adequate
- ☐ There are not enough course instructors for the number of participants
- ☐ There are not enough courses to enable all paediatricians/residents to participate
- ☐ There are no obstacles in my opinion
- ☐ Other (please specify)

52. What is, in your opinion, the optimal interval for retraining in paediatric and neonatal life support?

- ◯ Every 3 months
- ◯ Every 6 months
- ◯ Every year
- ◯ Every 2 years
- ◯ Other (please specify)

End of survey. Thank you very much for your contribution!
