# Supplementary material for: Training practices in neonatal and paediatric life support: A survey among healthcare professionals working in paediatrics
Source: Resusc Plus. 2021 Jan 6;5:100063. doi: 10.1016/j.resplu.2020.100063 (PMC8244515; doi:10.1016/j.resplu.2020.100063)
Supplement: Supplementary file 2 [file mmc2.pdf]

Electronic Supplementary Material 2

**Manuscript title**	Training practices in neonatal and paediatric life support: a survey among healthcare professionals working in paediatrics
**Journal**	Resuscitation Plus
**Corresponding author**	Mathijs Binkhorst, Radboud Institute for Health Sciences (RIHS), Department of Neonatology (804), Radboud University Medical Center Amalia Children's Hospital, P.O. Box 9101, 6500 HB, Nijmegen, the Netherlands, Tel: + 31 24 361 4430, Fax: + 31 24 361 64 28. Email: mathijs.binkhorst@radboudumc.nl

Survey announcement/invitational email

Dear colleague,

My name is Mathijs Binkhorst and I am a paediatrician-neonatologist in the Radboud University Medical Center in Nijmegen, the Netherlands. Recently, I contacted you by mail or phone about a large European survey study we are conducting among paediatricians and paediatric residents. In a few cases, I found your contact details on the Internet. I would like to ask you to distribute this email among **all paediatricians (incl. subspecialists) and paediatric residents** in your hospital. If possible, we would appreciate it if you can also send this mail to paediatric colleagues in affiliated hospitals in your region. Thank you in advance.

This survey is about **training and examination practices in neonatal and paediatric life support.** It is spread to hospitals in multiple countries in Europe. Our goal is to gain insight in

the implementation and application of neonatal and paediatric resuscitation guidelines and to evaluate how teaching, training, and testing of neonatal and paediatric life support actually take place in your country.

We kindly ask you to complete our survey, which will take approximately **10 minutes**. You can open the survey by clicking on the link below. All respondents and hospitals will remain anonymous. Data will only be reported per country.

https://www.surveymonkey.com/r/europeansurveypaediatricneonatallifesupport_radboudumc

We truly appreciate your valuable contribution to our research! We hope that many paediatricians and paediatric residents will fill in our survey, for it is an unprecedented attempt to gather this information on such a large scale.

On behalf of all co-workers,

Mathijs Binkhorst, MD, PhD student

Paediatrician-neonatologist

Radboud University Medical Center

Amalia Children's Hospital

Nijmegen, the Netherlands

Mathijs.Binkhorst@radboudumc.nl
