# Supplementary material for: Training practices in neonatal and paediatric life support: A survey among healthcare professionals working in paediatrics
Source: Resusc Plus. 2021 Jan 6;5:100063. doi: 10.1016/j.resplu.2020.100063 (PMC8244515; doi:10.1016/j.resplu.2020.100063)
Supplement: Supplementary file 3 [file mmc3.pdf]

Electronic Supplementary Material 3

**Manuscript title**	Training practices in neonatal and paediatric life support: a survey among healthcare professionals working in paediatrics
**Journal**	Resuscitation Plus
**Corresponding author**	Mathijs Binkhorst, Radboud Institute for Health Sciences (RIHS), Department of Neonatology (804), Radboud University Medical Center Amalia Children’s Hospital, P.O. Box 9101, 6500 HB, Nijmegen, the Netherlands, Tel: + 31 24 361 4430, Fax: + 31 24 361 64 28, Email: mathijs.binkhorst@radboudumc.nl

Table 2c. Responses (n=63) from other countries to items on PBLS, PALS, and NLS

**Item (n)** [1]	**Responses, n (%)**					
**Paediatric Basic Life Support**						
Do you receive PBLS training? (n=54)	≥1x/year<br>27 (50.0%)	<1x/year<br>20 (37.0%)	No/not yet<br>7 (13.0%)			
Does your hospital offer PBLS training? (n=54)	Yes<br>42 (77.8%)	No<br>11 (20.4%)	Don't know<br>1 (1.9%)			
PBLS training for all professionals caring for children in your hospital? (n=51)	Yes<br>11 (21.6%)	Most, not all<br>14 (27.5%)	P, PR, and PN<br>13 (25.5%)	P and PR<br>1 (2.0%)	Don't know<br>10 (19.6.%)	Other [2]<br>2 (3.9%)
Use of high or low-fidelity manikins? (n=51)	High<br>8 (15.7%)	Low<br>15 (29.4%)	Both<br>22 (43.1%)	Neither<br>1 (2.0%)	Don't know<br>5 (9.8%)	
Do you recertify for PBLS as needed? (n=55)	Yes<br>26 (47.3%)	No<br>6 (10.9%)	No guideline [3]<br>14 (25.5%)	Don't know<br>9 (16.4%)		
Have you read latest PBLS guideline? [4] (n=55)	Completely<br>39 (70.9%)	Partially<br>12 (21.8%)	No<br>4 (7.3%)			
Fully capable of performing PBLS instantly? (n=56)	Yes<br>40 (71.4%)	No<br>12 (21.4%)	Other [5]<br>4 (7.1%)			

**Paediatric Advanced Life Support**						
Do you receive PALS training? (n=50)	≥1x/year<br>17 (34.0%)	<1x/year<br>24 (48.0%)	No/not yet<br>9 (18.0%)			
Does your hospital offer PALS training? (n=48)	Yes<br>35 (72.9%)	No<br>11 (22.9%)	Don't know<br>2 (4.2%)			
Is this PALS training multidisciplinary? (n=44)	Yes<br>30 (68.2%)	No<br>11 (25.0%)	Don't know<br>3 (6.8%)			
Use of high or low-fidelity manikins? (n=43)	High<br>7 (16.3%)	Low<br>11 (25.6%)	Both<br>20 (46.5%)	Neither<br>1 (2.3%)	Don't know<br>4 (9.3%)	
Duration of latest national PALS course? (n=42)	1 day<br>19 (45.2%)	2 days<br>13 (31.0%)	3 days<br>8 (19.0%)	4 days<br>0 (0.0%)	Don't know<br>1 (2.4%)	Other [6]<br>1 (2.4%)
Hands-on time during latest PALS course? (n=43)	25%<br>2 (4.7%)	50%<br>18 (41.9%)	75%<br>19 (44.2%)	100%<br>1 (2.3%)	Don't know<br>3 (7.0%)	
Do you recertify for PALS as needed? (n=47)	Yes<br>17 (36.2%)	No<br>6 (12.8%)	No guideline [3]<br>14 (29.8%)	Don't know<br>10 (21.3%)		
Have you read latest PALS guideline? [4] (n=47)	Completely<br>33 (70.2%)	Partially<br>9 (19.1%)	No<br>5 (10.6%)			
Fully capable of performing PALS instantly? (n=47)	Yes<br>27 (57.4%)	No<br>15 (31.9%)	Other [7]<br>5 (10.6%)			

**Neonatal Life Support** [8]						
Do you receive NLS training? (n=45)	≥1x/year<br>18 (40.0%)	<1x/year<br>17 (37.8%)	No/not yet<br>10 (22.2%)			
Does your hospital offer NLS training? (n=41)	Yes<br>34 (82.9%)	No<br>7 (17.1%)	Don't know<br>0 (0.0%)			
Is this NLS training multidisciplinary? (n=39)	Yes<br>29 (74.4%)	No<br>7 (17.9%)	Don't know<br>3 (7.7%)			
Use of high or low-fidelity manikins? (n=39)	High<br>6 (15.4%)	Low<br>14 (35.9%)	Both<br>16 (41.0%)	Neither<br>1 (2.6%)	Don't know<br>2 (5.1%)	

Duration of latest national NLS course? (n=35)	1 day 23 (65.7%)	2 days 6 (17.1%)	3 days 1 (2.9%)	4 days 0 (0.0%)	Don't know 3 (8.6%)	Other [9] 2 (5.7%)
Hands-on time during latest NLS course? (n=37)	25% 4 (10.8%)	50% 12 (32.4%)	75% 15 (40.5%)	100% 1 (2.7%)	Don't know 5 (13.5%)	
Do you recertify for NLS as needed? (n=40)	Yes 14 (35.0%)	No 5 (12.5%)	No guideline [3] 10 (25.0%)	Don't know 11 (27.5%)		
Have you read latest NLS guideline? [4] (n=41)	Completely 28 (68.3%)	Partially 9 (22.0%)	No 4 (9.8%)			
Fully capable of performing NLS instantly? (n=41)	Yes 33 (80.5%)	No 8 (19.5%)	Other 0 (0.0%)			

Legend to Table 2c

NLS, neonatal life support; P, paediatricians; PALS, paediatric advanced life support; PBLS, paediatric basic life support; PN, paediatric nurses; PR, paediatric residents.

[1] Number of respondents for each item between parentheses; this number was obtained after subtracting skipped and inapplicable items from 63.

[2] Offered to all, but not compulsory; one invalid response.

[3] Recertification interval not specified in national guidelines.

[4] European Resuscitation Council guideline (2015) or national guideline.

[5] In doubt, mostly/probably.

[6] Invalid response.

[7] In doubt, partly, mostly/probably, after training, with supervision.

[8] Survey stated 'NLS or NALS', but Neonatal Advanced Life Support (NALS) courses had just started when this survey was conducted, so responses only pertained to NLS.

[9] Two invalid responses.

Table 2d. Responses (n=498) from all countries to items on PBLS, PALS, and NLS

Item (n) [1]	Responses, n (%)					
**Paediatric Basic Life Support**						
Do you receive PBLS training? (n=432)	≥1x/year 282 (65.3%)	<1x/year 101 (23.4%)	No/not yet 49 (11.3%)			
Does your hospital offer PBLS training? (n=419)	Yes 365 (87.1%)	No 32 (7.6%)	Don't know 22 (5.3%)			
PBLS training for all professionals caring for children in your hospital? (n=402)	Yes 126 (31.3%)	Most, not all 95 (23.6%)	P, PR, and PN 69 (17.2%)	P and PR 9 (2.2%)	Don't know 90 (22.4%)	Other [2] 13 (3.2%)
Use of high or low-fidelity manikins? (n=401)	High 47 (11.7%)	Low 183 (45.6%)	Both 126 (31.4%)	Neither 6 (1.5%)	Don't know 39 (9.7%)	
Do you recertify for PBLS as needed? (n=415)	Yes 267 (64.3%)	No 65 (15.7%)	No guideline [3] 28 (6.7%)	Don't know 55 (13.3%)		
Have you read latest PBLS guideline? [4] (n=419)	Completely 275 (65.6%)	Partially 72 (17.2%)	No 72 (17.2%)			
Fully capable of performing PBLS instantly? (n=420)	Yes 359 (85.5%)	No 41 (9.8%)	Other [5] 20 (4.8%)			

**Paediatric Advanced Life Support**						
Do you receive PALS training? (n=412)	≥1x/year 205 (49.8%)	<1x/year 144 (35.0%)	No/not yet 63 (15.3%)			
Does your hospital offer PALS training? (n=384)	Yes 287 (74.7%)	No 74 (19.3%)	Don't know 23 (6.0%)			
Is this PALS training multidisciplinary? (n=339)	Yes 223 (65.8%)	No 85 (25.1%)	Don't know 31 (9.1%)			
Use of high or low-fidelity manikins? (n=338)	High 45 (13.3%)	Low 133 (39.3%)	Both 114 (33.7%)	Neither 6 (1.8%)	Don't know 40 (11.8%)	
Duration of latest national PALS course? (n=345)	1 day 68 (19.7%)	2 days 66 (19.1%)	3 days 186 (53.9%)	4 days 3 (0.9%)	Don't know 5 (1.4%)	Other [6] 17(4.9%)
Hands-on time during latest PALS course? (n=343)	25% 8 (2.3%)	50% 114 (33.2%)	75% 180 (52.5%)	100% 9 (2.6%)	Don't know 32 (9.3%)	
Do you recertify for PALS as needed? (n=377)	Yes 257 (68.2%)	No 56 (14.9%)	No guideline [3] 27 (7.2%)	Don't know 37 (9.8%)		
Have you read latest PALS guideline? [4] (n=379)	Completely 241 (63.6%)	Partially 75 (19.8%)	No 63 (16.6%)			
Fully capable of performing PALS instantly? (n=381)	Yes 264 (69.3%)	No 80 (21.0%)	Other [7] 37 (9.7%)			

**Neonatal Life Support** [8]						
Do you receive NLS training? (n=394)	≥1x/year 209 (53.0%)	<1x/year 117 (29.7%)	No/not yet 68 (17.3%)			
Does your hospital offer NLS training? (n=371)	Yes 299 (80.6%)	No 46 (12.4%)	Don't know 26 (7.0%)			
Is this NLS training multidisciplinary? (n=341)	Yes 229 (67.2%)	No 71 (20.8%)	Don't know 41 (12.0%)			

Use of high or low-fidelity manikins? (n=337)	High<br>35 (10.4%)	Low<br>166 (49.3%)	Both<br>88 (26.1%)	Neither<br>4 (1.2%)	Don't know<br>44 (13.1%)	
Duration of latest national NLS course? (n=314)	1 day<br>258 (82.2%)	2 days<br>30 (9.6%)	3 days<br>1 (0.3%)	4 days<br>0 (0.0%)	Don't know<br>9 (2.9%)	Other [9]<br>16<br>(5.1%)
Hands-on time during latest NLS course? (n=319)	25%<br>16 (5.0%)	50%<br>111 (34.8%)	75%<br>146 (45.8%)	100%<br>9 (2.8%)	Don't know<br>37 (11.6%)	
Do you recertify for NLS as needed? (n=371)	Yes<br>196 (52.8%)	No<br>85 (22.9%)	No guideline [3]<br>32 (8.6%)	Don't know<br>58 (15.6%)		
Have you read latest NLS guideline? [4] (n=373)	Completely<br>257 (68.9%)	Partially<br>55 (14.7%)	No<br>61 (16.4%)			
Fully capable of performing NLS instantly? (n=374)	Yes<br>319 (85.3%)	No<br>41 (11.0%)	Other [10]<br>14 (3.7%)			

Legend to Table 2d

NLS, neonatal life support; P, paediatricians; PALS, paediatric advanced life support; PBLS, paediatric basic life support; PN, paediatric nurses; PR, paediatric residents.

[1] Number of respondents for each item between parentheses; this number was obtained after subtracting skipped and inapplicable items from 498.

[2] Emergency department nurses and specialists, paediatric surgeons, anaesthesiologists, residents in anaesthesiology, maternity/obstetric ward nurses, midwifes, gynaecologists, and residents in gynaecology.

[3] Recertification interval not specified in national guidelines.

[4] European Resuscitation Council guideline (2015) or national guideline.

[5] In doubt, 'on paper', partly, mostly/probably, insufficient clinical exposure to know, after training, with supervision.

[6] 1-day refresher course, 2-day refresher course, and several invalid responses.

[7] In doubt, 'on paper', partly, mostly/probably, insufficient clinical exposure to know, after training, with supervision, depending on situation/case, except for cardiac arrhythmia cases.

[8] Survey stated 'NLS or NALS', but Neonatal Advanced Life Support (NALS) courses had just started when this survey was conducted, so responses only pertained to NLS.

[9] All invalid responses.

[10] In doubt, 'on paper', partly, insufficient clinical exposure to know, after training, with supervision, except neonatal intubation.
